# Effects of paternal and chronological age on *BEGAIN* methylation and its possible role in autism

**DOI:** 10.18632/aging.205275

**Published:** 2023-11-28

**Authors:** Ramya Potabattula, Andreas Prell, Marcus Dittrich, Caroline Nava, Christel Depienne, Yosra Bejaoui, Nady El Hajj, Thomas Hahn, Martin Schorsch, Thomas Haaf

**Affiliations:** 1 Institute of Human Genetics, Julius Maximilians University, Würzburg, Germany; 2 Department of Bioinformatics, Julius Maximilians University, Würzburg, Germany; 3 U.F. de Neurogénétique Moléculaire et Cellulaire, Dpt. de Génétique et Cytogénétique, Groupe Hospitalier Pitié-Salpêtrière, Paris, France; 4 Institute of Human Genetics, University Hospital Essen, Essen, Germany; 5 College of Health and Life Sciences and College of Science and Engineering, Hamad Bin Khalifa University, Doha, Qatar; 6 Fertility Center, Wiesbaden, Germany

**Keywords:** age and sex effect, autism spectrum disorder, BEGAIN, chronological aging, paternal age effect, sperm methylation

## Abstract

Children from old fathers carry an increased risk for autism spectrum (ASD) and other neurodevelopmental disorders, which may at least partially be mediated by paternal age effects on the sperm epigenome. The brain enriched guanylate kinase associated (BEGAIN) protein is involved in protein-protein interactions at and transmission across synapses. Since several epigenome-wide methylation screens reported a paternal age effect on sperm *BEGAIN* methylation, here we confirmed a significant negative correlation between *BEGAIN* promoter methylation and paternal age, using more sensitive bisulfite pyrosequencing and a larger number of sperm samples. Paternal age-associated *BEGAIN* hypomethylation was also observed in fetal cord blood (FCB) of male but not of female offspring. There was no comparable maternal age effect on FCB methylation. In addition, we found a significant negative correlation between *BEGAIN* methylation and chronological age (ranging from 1 to 70 years) in peripheral blood samples of male but not of female donors. *BEGAIN* hypomethylation was more pronounced in male children, adolescents and adults suffering from ASD compared to controls. Both genetic variation (CC genotype of SNP rs7141087) and epigenetic factors may contribute to *BEGAIN* promoter hypomethylation. The age- and sex-specific *BEGAIN* methylation trajectories in the male germ line and somatic tissues, in particular the brain, support a role of this gene in ASD development.

## INTRODUCTION

The trend towards delayed parenthood due to economic and/or social reasons has been constantly increasing [1]. Advanced parental age is not only associated with a growing demand for prenatal diagnostic testing and assisted reproductive technologies, but also with an elevated risk for medical problems in offspring. It is known for a long time that the oocyte aneuploidy rate increases with maternal age, causing fertility problems, spontaneous abortions, and children with Down syndrome [2]. In contrast, the influence of paternal age on reproduction and offspring health is usually underestimated. Despite life-long spermatogenesis and fertility, the developmental potential of sperm declines with paternal age [3], whereas the offspring risk for some rare monogenic disorders such as achondroplasia and neurofibromatosis type 1 [4] as well as for multifactorial neurodevelopmental disorders including autism and schizophrenia is increasing [5, 6]. The higher number of spermatogonial cell divisions and de novo genetic mutations [4, 7] explains only a percentage of the increased disease susceptibility (i.e., for monogenic disorders) in the offspring of aging men. Assuming that the error rate of the copying process during DNA replication is 10 to 100-fold higher for the epigenome than for the genome [8], the increased mutational burden in sperm from older men may be largely due to epimutations, rather than DNA sequence mutations. Mouse studies have linked paternal age-associated changes in sperm DNA methylation with alterations in brain gene methylation/expression and abnormal behavior in the offspring [9, 10], providing a mechanism for transgenerational epigenetic effects. Previously we have shown shared epigenetic signatures between sperm of aging fathers and cord blood of resulting offspring in humans [11]. However, the long generation time and limited access to the critical tissues renders studies on transgenerational inheritance in humans difficult. The human sperm epigenome is affected by stochastic and various environmental factors, including fertility status, diet, and aging [12, 13].

Several epigenome-wide association studies have identified a large number (>2,000) of genes with age-associated differentially methylated regions (ageDMRs) in sperm [14–19]. Although overall there was little overlap between different data sets, sperm ageDMRs appear to be enriched in genes involved in embryonic and neuronal development [14–19]. This argues in favor of the notion that paternal age effects on the sperm epigenome contribute to the increased disease risk for neurodevelopmental disorders in the offspring. So far, only 40 genes with sperm ageDMRs have been replicated in at least three independent genome-wide methylation screens [19], which makes them primary candidates for mediating paternal age effects on the next generation. Here, we focused on one of these top candidates, the *BEGAIN* promoter region. The observation that *BEGAIN* is hypomethylated in the brain of children with autism spectrum disorder (ASD), compared to controls [20], provides additional evidence for its role in the etiopathogenesis of neurodevelopmental disorders. The human *BEGAIN* gene encodes the brain-enriched guanylate kinase-associated protein, which may sustain the postsynaptic density (PSD) structure and be involved in the regulation of synaptic functions. Promoter methylation is critical for shaping gene expression during differentiation and development [21, 22]. It is tempting to speculate that transmission of paternal age-associated sperm methylation changes into the next generation modulates *BEGAIN* regulation and susceptibility to neurodevelopmental disorders.

## RESULTS

### Paternal age effect on sperm *BEGAIN* methylation

**Figure 1:**
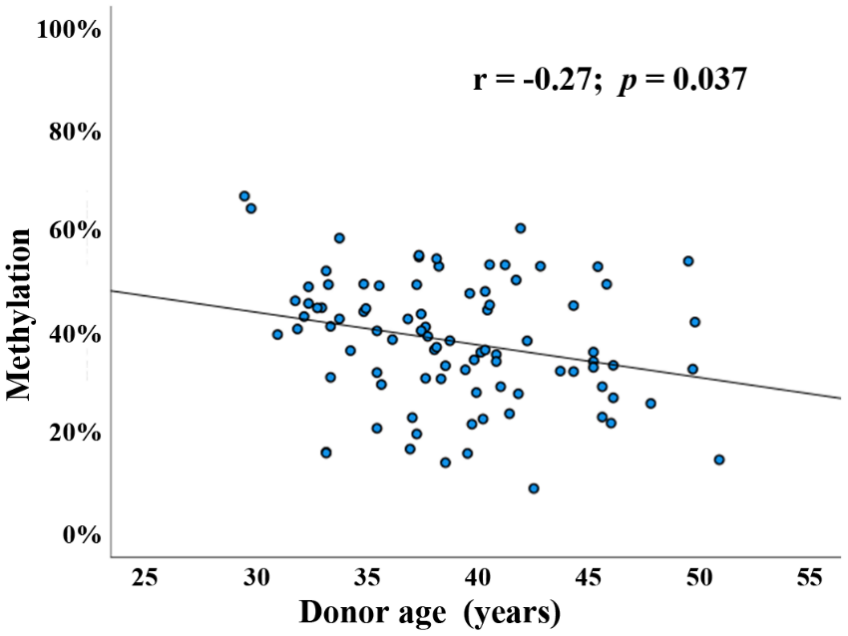
**Paternal age effect on *BEGAIN* promoter methylation in human sperm.** Scatter plot showing significant (Pearson’s r = -0.27; *p* = 0.037) negative correlation between donor age (x-axis in years) and mean methylation of the 9 analyzed CpGs (y-axis in %) in 90 human sperm samples (blue dots).

Several genome-wide methylation screens using Illumina arrays [14, 18] and reduced representation bisulfite sequencing (RRBS) [19] suggested that *BEGAIN* methylation in sperm is dependent on donor age. For more accurate quantification of the average methylation level of the *BEGAIN* promoter region, we designed a bisulfite pyrosequencing (BPS) assay covering 9 adjacent CpG sites (Supplementary Table 1). The methylation difference between technical replicates (including bisulfite conversion) is in the order of one percentage point (data not shown). Using this assay, *BEGAIN* promoter methylation was determined in 90 normozoospermic sperm samples from couples attending a fertility center. Donor age ranged from 29 to 51 years (mean ± SD; 38.9 ± 4.9), body mass index (BMI) from 19 to 32 kg/m^2^ (25.5 ± 2.9), and sperm concentration from 15 to 260 million/ml (86.6 ± 45.5). Since it is usually the density of methylated CpGs rather than the individual CpGs in the promoter region that turns a gene on or off [22], we determined average methylation of all 9 CpGs in the target region. Pearson’s partial correlations were implemented to adjust for the potential confounding factors such as sperm concentration and donor BMI. Mean methylation of the *BEGAIN* promoter region in human sperm displayed a significant negative correlation (Pearson’s r = -0.27; *p* = 0.037) with donor age (Figure 1). Moreover, methylation of each analyzed individual CpG was significantly negatively correlated with donor age (data not shown).

### *BEGAIN* methylation in fetal cord blood samples

**Figure 2:**
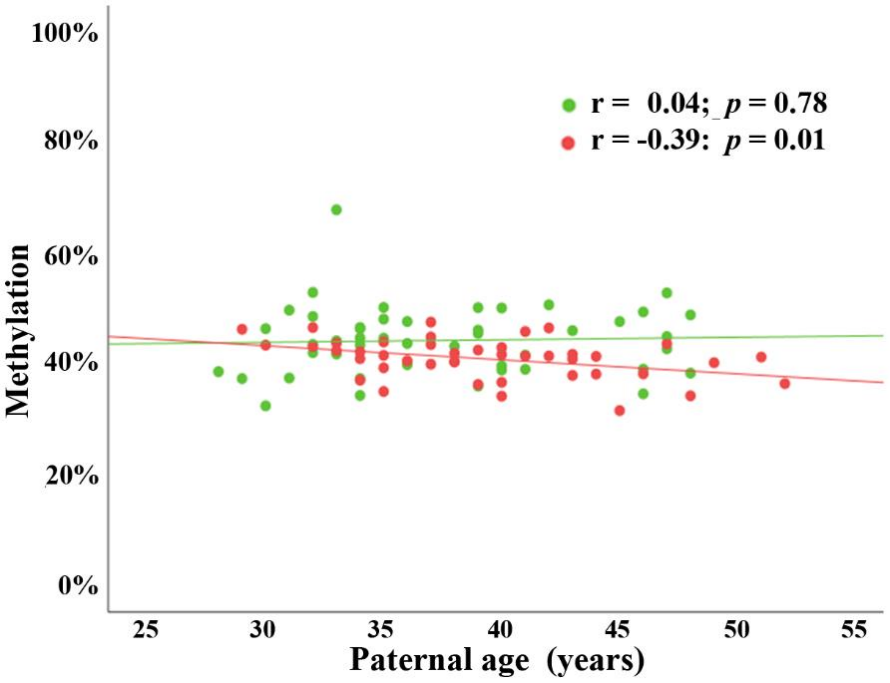
**Paternal age effect on *BEGAIN* promoter methylation in male but not in female cord bloods.** Scatter plot showing significant negative correlation (Pearson’s r = -0.39; *p* = 0.01) between paternal age (x-axis in years) and mean methylation of 9 CpGs (y-axis in %) in 43 male FCBs (red dots). There was no paternal age effect (Pearson’s r = 0.04; *p* = 0.78) in 46 female FCBs (green dots).

To study possible transmission of age-associated changes in sperm *BEGAIN* methylation into the next generation, we carried out BPS in fetal cord bloods (FCBs) of 89 children (46 females and 43 males) conceived by assisted reproduction. Paternal age ranged from 28 to 52 years (mean ± SD; 38.3 ± 5.7) and maternal age from 22 to 42 years (33.9 ± 4.1). Paternal age negatively correlated with *BEGAIN* methylation, both at regional and individual CpG levels, in male (Pearson’s r = -0.39; *p* = 0.01), but not in female cord bloods (Pearson’s r = 0.04; *p* = 0.78) (Figure 2). Maternal age did not correlate with *BEGAIN* methylation in both male (Pearson’s r = -0.10; *p* = 0.52), and female FCBs (Pearson’s r = -0.06; *p* = 0.68) (Supplementary Figure 1).

BPS measures average methylation in a mixture of millions of maternal and paternal DNA molecules in a genomic DNA sample. To study paternal age effects on the next generation, it would be desirable to distinguish between paternal and maternal alleles in the offspring’s epigenome. To this end, we developed a BPS genotyping assay for SNP rs7141087 (minor allele frequency in Europeans 0.39; C) in the *BEGAIN* promoter region. By analyzing informative FCBs and corresponding fathers’ sperm samples, we identified a total of 38 heterozygous FCBs (16 females and 22 males) with homozygous fathers. Deep bisulfite sequencing (DBS) is an amplicon-based single molecule sequencing technique that can distinguish between individual (parental) alleles. A DBS assay covering SNP rs7141087 and 14 CpGs (including the 9 CpGs analyzed by BPS) allowed us to determine methylation of the paternal allele in informative FCB samples. A regression model was fitted to the data adjusting for the effect of the SNP base (T/C) on allele methylation. Although due to small sample size (14 male FCBs with an T and 8 with a C allele) the results did not reach significance (coefficient of regression model = -0.09; *p* = 0.62), we observed a negative association of paternal allele methylation with paternal age in male FCBs (Supplementary Figure 2).

*BEGAIN* has been reported as an imprinted gene in mouse, bovine, and sheep (https://www.geneimprint.com/site/genes/Mus_musculus_Begain), however so far there is no evidence for imprinting in humans. The *BEGAIN* promoter studied here by DBS showed very similar methylation levels of paternal and maternal alleles in FCBs (Supplementary Figure 3). Moreover, sperm methylation in the medium range (20-60%) measured by BPS (Figure 1) is not compatible with imprinting.

### *BEGAIN* methylation in male peripheral blood samples is decreasing with chronological age

**Figure 3:**
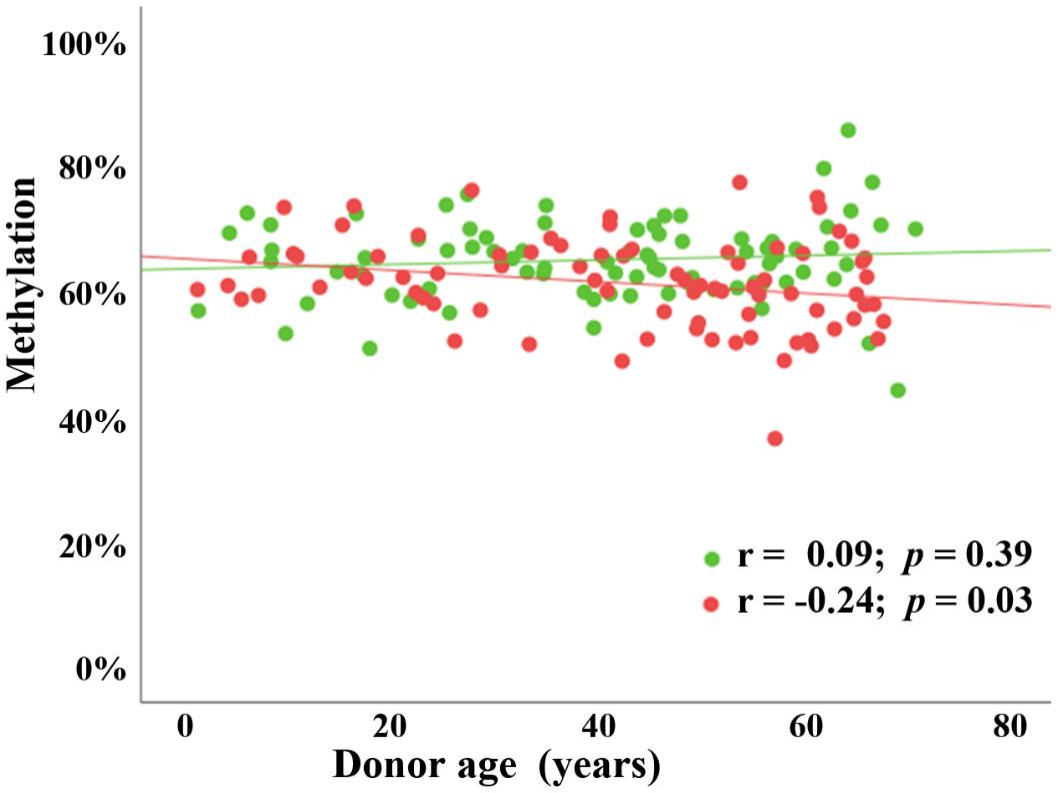
**Sex-specific (chronological) age effect on *BEGAIN* promoter methylation in peripheral blood samples.** Scatter plot showing significant negative correlation (Pearson’s r = -0.24; *p* = 0.03) between chronological donor age (x-axis in years) and mean methylation (y-axis in %) in 82 male blood samples (red dots) using BPS. There was no detectable age effect (Pearson’s r = 0.09; *p* = 0.39) in 80 female blood samples (green dots).

The paternal age effect on FCB methylation may be concealed by a much larger effect of chronological aging of an individual’s organs and tissues. To demonstrate this, we determined methylation levels of the *BEGAIN* promoter in peripheral blood samples of 82 males and 80 females, ranging from 1 to 70 years in age. There was a significant negative correlation, both at regional and individual CpG level, between donor age and *BEGAIN* methylation in male peripheral blood samples (Pearson’s r = -0.24; *p* = 0.03), but interestingly not in the females (Pearson’s r = 0.09; *p* = 0.39) (Figure 3). In males, the age effect on peripheral blood samples (r = -0.24) was similar to that observed in sperm (r = -0.27), suggesting that it may occur in all tissues and cell types of the male body, including the germ line.

Since age effects on *BEGAIN* methylation were only observed in males, we wanted to find out whether this gene also shows sex-specific expression patterns. According to the genotype-tissue expression (GTEx) portal (https://gtexportal.org) *BEGAIN* is widely expressed in different regions of the brain (with the highest expression in cerebellum and cerebellar hemisphere) and to a lesser extent in many other tissues (Supplementary Figure 4). Interestingly, in most brain regions *BEGAIN* expression was somewhat higher in males, compared to females. For example, the median transcripts per million (TPM) in the cerebellum was 76.2 in males (n = 174), compared to 68.5 in females (n = 67).

### The male age effect on *BEGAIN* methylation may be exaggerated in autistic individuals

**Figure 4:**
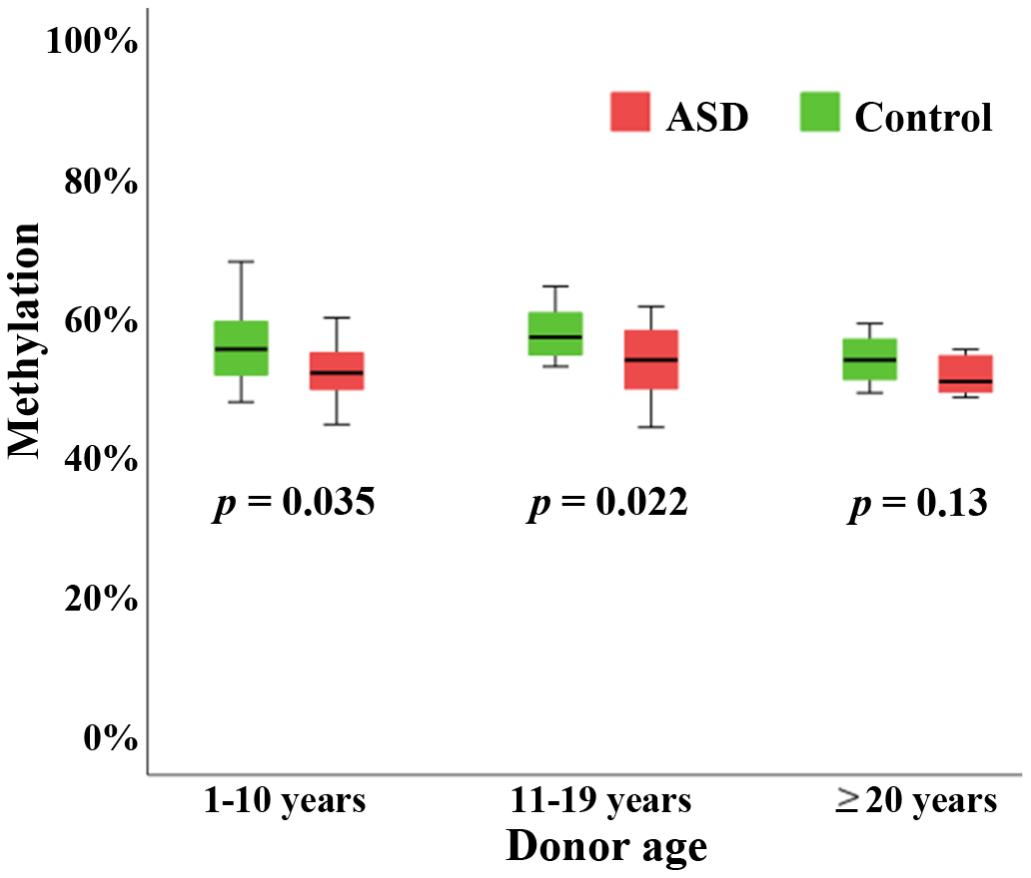
**Hypomethylation of the *BEGAIN* promoter in peripheral blood samples of autistic individuals.** Box plots showing methylation (y-axis in %) of the human *BEGAIN* promoter in peripheral blood of individuals with ASD (red color), compared to controls (green color). *BEGAIN* was hypomethylated in autistic samples in all three age groups, consisting of 20 matched pairs ≤ 10 years, 18 pairs between 11 and 19 years, and 8 pairs ≥ 20 years, respectively. In the two younger groups the between-group difference was significant. The median is presented by a horizontal line. The bottom of the box indicates the 25^th^ and the top the 75^th^ percentile.

To test our hypothesis that *BEGAIN* plays a role in neurodevelopment and its disorders, we compared *BEGAIN* methylation in the peripheral blood samples of 46 males with ASD versus 46 age- and sex-matched controls. The age of the matched pairs ranged from 2 to 43 years (mean ± SD; 12.7 ± 9.0). The Mann-Whitney *U*-test revealed a highly significant (*p* < 0.001) hypomethylation of the *BEGAIN* promoter in autistic individuals. To demonstrate differences in postnatal *BEGAIN* methylation trajectories between autistic and non-autistic individuals, we classified samples into three age groups. Twenty autistic-control pairs were ≤ 10 years, 18 pairs between 11 and 19 years, and 8 pairs ≥ 20 years old. *BEGAIN* was significantly hypomethylated in autistic individuals ≤ 10 years (Mann-Whitney *U*-test; *p* = 0.035) and in the group from 11 to 19 years (*p* = 0.022), compared to controls (Figure 4). *BEGAIN* hypomethylation was also observed in the group ≥ 20, but due to small sample size did not reach significance.

The observed *BEGAIN* hypomethylation in the ASD group appears to be a combined genetic and epigenetic effect. Of the 46 individuals with ASD, 12 displayed a TT, 18 a CT, and 16 a CC genotype for SNP rs7141087. In contrast, of the 46 control individuals 20 were endowed with a TT, 22 a CT and 4 a CC genotype (Supplementary Figure 5). Regression analysis revealed a significant effect of the SNP base, with a decrease in methylation for the CT (-2.5%, *p* = 0.01) and the CC (-5.7%, *p* = 3.72e-06) genotype. Since individuals with CC were more frequent in the ASD and individuals with TT in the control group, this SNP partially explains *BEGAIN* hypomethylation in our ASD group. Nevertheless, following adjustment for the SNP effect, there was an additional significant decrease of overall methylation in the ASD group (-2.3%, *p* = 0.008), compared to controls.

**Figure 5:**
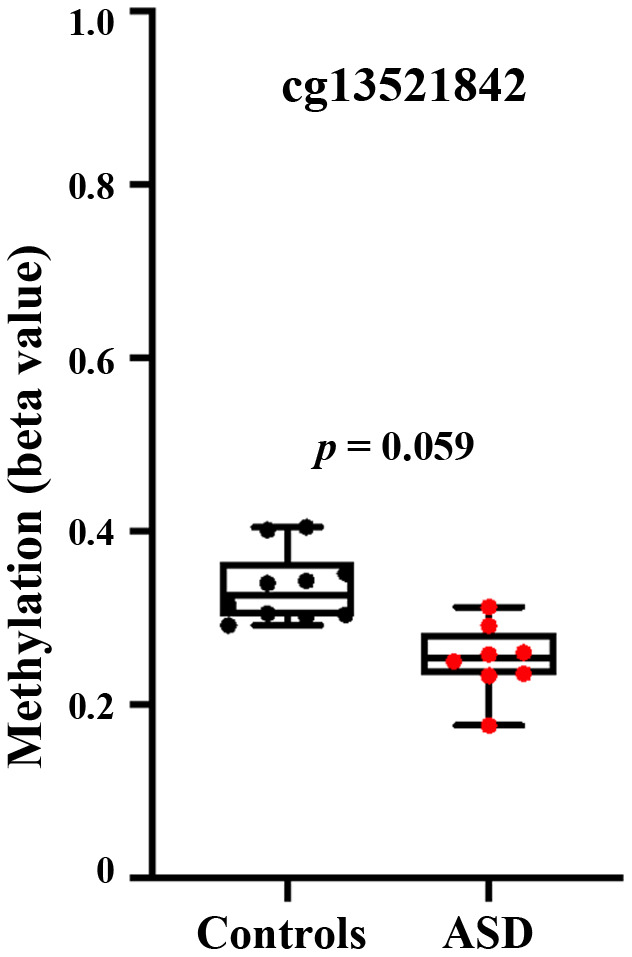
**Hypomethylation of the *BEGAIN* promoter CpG 13521842 in autistic brain.** Re-analysis of genome-wide Infinium 450K human methylation array data [20], using GEO2R tool in male brain samples. The box plots reveal hypomethylation (*p* = 0.059) in the anterior cingulate cortex of 8 male autistic individuals, compared to 10 controls.

Our results on peripheral blood samples are corroborated by a methylation array screen [20], reporting hypomethylation of the *BEGAIN* locus in anterior cingulate cortex of individuals with ASD, compared to controls. One (cg13521842) of two significant CpG sites in this study lies in the target region of our BPS assay. Since the published results were based on male and female brain samples, we reanalyzed the data using GEO2R tool, including only the male (8 autistic and 10 control) samples in our analysis. CpG 13521842 showed a beta value of 0.25 in ASD and 0.34 in control samples (Figure 5), however due to the small sample this 9% methylation difference between groups was not significant. The *p*-value was slightly higher than 0.05 (*p* = 0.059).

## DISCUSSION

The paternal age effect on the human sperm methylome has been extensively studied using Illumina methylation arrays [14, 18], whole genome bisulfite sequencing (WGBS) [17], methyl capture sequencing [15], and RRBS [16, 19]. Moreover, several epigenetic clocks for sperm donor age prediction were derived by linear regression algorithms on different genome-wide DNA methylation data sets [15, 17, 23, 24]. The surprisingly low overlap between the identified sperm ageDMRs in different studies may be partially explained by the differences in techniques, donor groups, and sample size. In a recent meta-analysis [19], we identified 241 genes (of 2,355 reported genes with sperm ageDMRs) which have been replicated in at least two independent genome-wide methylation screens. A common approach to interpreting changes in large gene lists is functional enrichment analysis. Consistent with earlier studies [14–18], these replicated candidate genes were associated with nervous system development, neurons, and synapses and, therefore, are primary candidates for mediating paternal age effects on neurodevelopment and behaviour of the offspring [19].

One important caveat remains. The reported age-associated methylation changes were usually (very) small and there was considerable overlap in methylation variation between sperm samples from old versus young males. When looking at individual loci, such as *BEGAIN*, it is hard to tell what are the biological effects of methylation changes in the order of a few percentage points. If any, their impact on *BEGAIN* regulation must be small. Published gene expression data in the GTEx portal suggest that *BEGAIN* expression in the brain, in particular the cerebellum and the cerebellar hemisphere, is about 10% higher in males, compared to females. Unfortunately, no computational method can be applied for proper between-sample normalization across the diverse set of tissues in GTEx. The gene expression visualizations therefore provide a qualitative measure of relative expression. Nevertheless, they support the idea that sex-specific *BEGAIN* methylation trajectories contribute to sexually dimorphic traits.

Similar to genetic variants identified in genome-wide association studies, sperm ageDMRs may help to uncover genes, pathways and/or mechanisms that are susceptible to aging. Sperm aging and the associated medical problems in the resulting embryos and offspring are multifactorial processes, involving stochastic and environmentally induced, genetic and epigenetic changes in numerous genes and pathways. In this light, multiple changes of small effect size rather than highly penetrant epimutations in a single or a few genes may contribute to paternal age effects.

### Paternal and chronological age effect on *BEGAIN* methylation in males

One of the top candidates from our previous meta-analysis [19] is a sperm ageDMR in the *BEGAIN* promoter region. First, we confirmed age-associated hypomethylation of *BEGAIN* in a larger number of normozoospermic (according to the 5^th^ edition of the WHO laboratory manual) sperm samples from couples wishing to have children. Although we cannot rule out the formal possibility that our findings are restricted to sperm from subfertile males and children conceived by assisted reproductive technologies, similar paternal age effects are likely to occur in fertile males and their offspring. To exclude somatic cell contamination, all sperm samples were purified by both the swim-up method and density gradient centrifugation. Moreover, all samples exhibited the appropriate parent-specific methylation patterns of the paternally imprinted *GTL2* and the maternally imprinted *PEG3* gene (data not shown). Following adjustment for potential confounding factors such as sperm concentration and donor BMI, the *BEGAIN* promoter region showed a significant negative correlation with donor age. This is consistent with our previous RRBS analysis [19], where the majority (74%) of genes with sperm ageDMRs became hypomethylated with age. An earlier study based on methylation arrays [14] even presented 95% hypomethylated sperm ageDMRs [14]. However, in other studies [15–18], genes with hypermethylated sperm ageDMRs predominated. Ribosomal DNA and other repeat DNA families also gained sperm methylation with age [25]. Collectively, these results suggest that both hypomethylation and hypermethylation occur during sperm aging at specific loci and regions of the genome.

Consistent with transgenerational inheritance, hypomethylation of the paternal *BEGAIN* allele was also detectable in FCBs of the resulting offspring. The mechanism by which acquired germ line methylation patterns can be transmitted to the next generation remains to be elucidated. Accumulating evidence suggests that some non-imprinted loci may also be resistant to postzygotic methylation reprogramming after fertilization or, if the marks are completely erased may be re-established [26, 27]. When looking at published WGBS data sets from human 2-cell, 4-cell, 8-cell, and morula stage embryos [28], we identified a locus on chromosome 14 (chr14:100,530,200-100,530,800 bp; hg38), which is located only 6.4 kb proximally from the *BEGAIN* gene (chr14:100,537,147-100,587,417 bp) and retains a certain methylation percentage after fertilization. Moreover, *BEGAIN* marks the centromeric boundary of the reciprocally imprinted *DLK1*-*GTL2* gene cluster [29], lying approximately 140 kb upstream of the paternally expressed *DLK1* (chr14:100,725,705-100,738,224) gene. The *BEGAIN* promoter studied here (chr14:100,569,573-100,570,032) resides approximately 240 kb upstream of the intergenic germ line DMR (chr14:100,809,097-100,809,494 bp) controlling *DLK1*-*GTL2* imprinting. *BEGAIN* appears to be subject to maternal imprinting in mouse, bovine and sheep (https://www.geneimprint.com/site/genes/Mus_musculus_Begain), generating paternally expressed transcripts in a tissue- and promoter-specific manner [30]. However, so far there is no evidence for imprinting in humans. The promoter region studied here exhibited medium methylation in sperm and no methylation difference between parental alleles in FCBs. We propose that *BEGAIN* has lost imprinting in the human lineage but may still be resistant to postzygotic reprogramming. Of course, since we have only looked at 500 bp in the main promoter we cannot exclude the possibility of imprinting in other regulatory regions of the *BEGAIN* locus.

By using BPS on a larger number of (46 female and 43 male) FCBs we found that the *BEGAIN* promoter becomes significantly hypomethylated with paternal age in male but not in female offspring. Similar sex-specific transgenerational effects have been observed before [13, 31]. Since BPS cannot distinguish between maternal and paternal DNA molecules, we developed a DBS assay including a SNP in the target region, to evaluate paternal allele methylation in the offspring. Since it is well-known that SNPs can influence DNA methylation [12], we used regression models to adjust for the cis-regulatory base pair effect. Due to the low number of informative male FCB samples, the observed *BEGAIN* hypomethylation of the paternal allele did not reach significance.

Indeed, the effect of paternal age on *BEGAIN* methylation in FCBs may be covered up by chronological age-associated methylation changes occurring in the offspring itself. BPS on peripheral blood samples covering an age range from 1 to 70 years revealed a significant age-associated hypomethylation of the *BEGAIN* promoter in males but not in females. This promotes the idea that paternal-age associated *BEGAIN* methylation changes in the male offspring are more important in early development, i.e., during embryogenesis, than later in life, when chronological age-associated changes have accumulated. The older the individual the bigger the impact of ontogenetic changes. Similar to *FOXK1* [11], the observed paternal age effects on *BEGAIN* methylation in sperm and FCB, respectively, were of small effect size.

### Sex-specific aging trajectories

Our study provides strong evidence for a sex-specific effect of both paternal age and chronological age of the offspring on *BEGAIN* methylation. In an epigenome-wide association study based on Illumina methylation arrays, 52 autosomal CpG sites (in 34 genes, including *BEGAIN*) and 597 X-linked CpGs (in 217 genes) showed significant and replicated age-by-sex methylation differences in peripheral blood [32]. The excess of X-linked genes with sex-specific aging trajectories may be at least partially due to their mosaic methylation state in females and increased skewing of X inactivation with age [33]. Genes subject to sex-specific aging appear to be enriched among downregulated genes in the testes and upregulated genes in the brain [32]. It seems plausible to assume that such sex-specific aging trajectories affecting the endocrine system, brain structure and function are an important mechanism underlying sexually dimorphic traits. It is well known that males and females differ in their susceptibilities to various neurodevelopmental and age-related pathologies as well as in their life expectancies [34]. The epigenetic clock appears to tick faster in males than in females [35, 36].

### Possible role for *BEGAIN* in autism

Autism is characterized by deficits in communication and social interaction as well as stereotypical behaviors. ASD has a strong and complex genetic component with up to 1,000 genes potentially implicated and a meta-analytic heritability estimate of 60–90% [37]. Environmental factors modulating cell signaling, metabolic, immune, and epigenetic processes can influence the development of ASD in genetically susceptible individuals [38]. Accumulating evidence supports brain inflammation and abnormal immune function (i.e. cytokine dysregulation, anti-brain antibodies) in autistic brains [39]. Like many other neurodevelopmental disorders, autism shows a strong sex bias, with four times more affected males than females [40, 41]. On the other hand, the rate of diagnosis and the presentation of these disorders may be skewed towards diagnosing males. Females may be underdiagnosed or present different symptoms. Both an increased disease susceptibility in males and protective effects in females have been proposed. The “extreme male brain theory of autism” which associates ASD with an exaggeratedly male-typical brain [42] has been under great scrutiny. Consistent with the “female protective model”, females with autism (and other neurodevelopmental disorders) display a higher mutational burden than males [43, 44].

Although overall little is known about the *BEGAIN* gene (20 PubMed entries up to October 2023), several lines of evidence support its role in ASD pathogenesis. *BEGAIN* is broadly expressed in the brain and located in dendrite, nucleus, and postsynapse of neurons. It interacts with the PSD95/SAP90 protein [45], a synaptic membrane-associated guanylate kinase attached to the postsynaptic membrane. *BEGAIN* may be involved in the regulation of postsynaptic neurotransmitter receptor activity, acting upstream of or within evoked excitatory postsynaptic potential (https://www.ncbi.nlm.nih.gov/gene/57596). The first experimental evidence comes from a methylation array screen, reporting the *BEGAIN* locus to be hypomethylated in the brain of individuals with autism [20]. Since this study did not consider the sex effect, we re-analyzed the published data set and confirmed hypomethylation of a promoter CpG (covered by our BPS assay) in male autistic brains.

In addition, we found *BEGAIN* hypomethylation in peripheral blood of male children, adolescents and adults with autism, compared to controls. The observed *BEGAIN* hypomethylation in our ASD group appears to be a combined genetic and epigenetic effect. Individuals with CC genotype of SNP rs7141087 which show a 6% lower methylation than the TT genotype are significantly more frequent in our ASD group than in controls. This could be due to an association of the C allele with autism. However, since our ASD cohort from France includes up to 10% samples from North Africans, compared to 1% in the control group, we cannot exclude that the abundance of individuals with CC genotype in our ASD group is mainly due to geographic/ethnic origin. The frequency of the C allele is considerably higher in Africans (86%) than in Europeans (39%). However, even after adjusting for the SNP effect, there still is a significant difference between ASD and controls.

## CONCLUSIONS

*BEGAIN* is one of only several dozen known autosomal genes with sex-specific methylation trajectories during ontogeny (chronological aging), which may contribute to shaping sexually dimorphic traits and disease susceptibilities. The male-specific hypomethylation of the *BEGAIN* promoter in blood, and by extrapolation other somatic tissues is exaggerated in males suffering from autism. Moreover, our results also show a paternal age effect on *BEGAIN* methylation in sperm and the male offspring (FCB). Epidemiological studies demonstrate an elevated risk for ASD in children from older fathers [5, 6, 46]. ASD and other complex psychiatric disorders associated with advanced paternal age manifest when adverse genetic, epigenetic, and/or environmental factors (all with small effect sizes) exceed a critical threshold. However, the functional implications of small age-associated methylation changes in *BEGAIN* in a multifactorial disease model remain to be elucidated.

## MATERIALS AND METHODS

### Study samples

The study on human sperm, fetal cord blood, and peripheral blood samples was approved by the ethics committee at the medical faculty of the University of Würzburg (no. 117/11 and 212/15). Written informed consent was collected from all the participants. The left-over swim-up sperm fractions (excess material) after *in vitro* fertilization (IVF) or intracytoplasmic sperm injection (ICSI) were collected at the Fertility Center Wiesbaden, pseudonymized, and frozen at -80° C until further use. After thawing, the swim-up sperm samples were purified further by silane-coated density gradients PureSperm 80 and 40 (Nidacon, Mölndal, Sweden). All analyzed FCB samples were from newborns conceived through IVF/ICSI in a single fertility center and were collected by collaborating obstetric clinics throughout Germany. Peripheral blood DNAs from mutation-negative individuals with an age range from 1 to 70 years were anonymized excess materials from predictive genetic diagnostics. Written informed consent was obtained for all controls. The peripheral blood samples from autistic patients were obtained from the Centre de Référence Déficiences Intellectuelles de Causes Rares and the Centre de Diagnostic Autisme, Pitié-Salpêtrière Hospital (Paris, France). Index cases were evaluated by specialized geneticists, pediatric neurologists and/or child psychiatrists. Patients were assessed with the Autism Diagnostic Interview-Revised (ADI-R). Informed consent was obtained and studies were approved by local ethics committees.

### Genomic DNA isolation and sodium bisulfite conversion

The purified human sperm samples were resuspended in 300 μl buffer. The stock buffer consisted of 5 ml of 5 M NaCl, 5 ml of 1 M Tris-HCl (pH 8), 5 ml of 10% SDS (pH 7.2), 1 ml of 0.5 M EDTA (pH 8), 1 ml of 100% β-mercaptoethanol, and 33 ml of dH_2_O. Upon addition of 100 μl (20 mg/ml; 600 mAU/ml) of proteinase K (Qiagen, Hilden, Germany), the samples were initially incubated for 2 h at 56° C on a thermomixer. Additional 20 μl proteinase K was added and samples were incubated for another 2 h at 56° C. Subsequently, human sperm DNA was isolated using the DNeasy Blood and Tissue Kit (Qiagen) following the manufacturer’s recommendations. For blood samples, DNA was isolated by the classical salting-out method. DNA concentration and purity were measured with a NanoDrop 2000c spectrophotometer (Thermo Scientific, MA, USA). Bisulfite conversion of DNA was performed using the EpiTect Fast 96 Bisulfite kit (Qiagen) and the converted DNA was stored at -20° C until further use.

### Bisulfite pyrosequencing

PCR and pyrosequencing primers (Supplementary Table 1) for the human *BEGAIN* promoter region were designed using the PyroMark Assay Design 2.0 software (Qiagen). DNA methylation standards with 0%, 50%, and 100% methylation were used for the assay establishment and validation. Fully methylated and unmethylated DNA standards (Qiagen) were used as controls in each run. PCR was carried out in a 25 μl reaction consisting of 2.5 μl 10x PCR buffer with MgCl_2_, 0.5 μl (10 mM) dNTPs, 1.25 μl (10 pmol/ml) of each forward and reverse primer, 0.2 μl (5 U/μl) FastStart Taq DNA polymerase (Roche Diagnostics, Mannheim, Germany), 1 μl (~25 ng) bisulfite converted DNA, and 18.3 μl ddH_2_O. Amplifications were performed with an initial denaturation at 95° C for 5 min, 35 cycles of 95° C for 30s, primer-specific annealing temperature of 60° C for 30s, and 72° C for 45s, and a final extension step at 72° C for 10 min. Pyro Q-CpG software (Qiagen) and PyroMark Gold Q96 CDT reagent kit (Qiagen) were used for pyrosequencing on the PyroMark Q96 MD system (Qiagen).

### Deep bisulfite sequencing

To distinguish alleles in informative samples, SNP rs7141087 was used, because it had the highest minor allele frequency (39%) in the *BEGAIN* promoter region. Primers for genotyping (Supplementary Table 1) on bisulfite converted DNA were designed using PyroMark Assay Design 2.0 software. Genotyping was performed on PyroMark Q96 MD system.

DBS primers (Supplementary Table 1) for the *BEGAIN* amplicon were used for the first-round PCR in 50 μl reactions consisting of 5 μl 10x PCR buffer with MgCl_2_, 1 μl (10 mM) of PCR grade nucleotide mixture, 2.5 μl (10 pmol/ml) of forward and reverse primers, 0.4 μl (5 U/μl) FastStart Taq DNA polymerase, 2 μl (~50 ng) of bisulfite-converted genomic DNA, and 36.6 μl ddH_2_O. Artificially demethylated (0%) and fully methylated (100%) DNAs served as controls for assessing the reliability of methylation measurements for each DBS amplicon. PCR products were cleaned with Agencourt AMPure XP beads (Beckmann Coulter, Krefeld, Germany), and quantified using the Qubit dsDNA BR assay system kit (Invitrogen, Karlsruhe, Germany), and diluted to a concentration of 0.2 ng/μl. In a second PCR, the samples from different assays were pooled together and barcoded using 48 multiplex identifiers (MIDs). NEBNext Multiplex Oligos for Illumina (Dual Index Primers Set 1) were used for the final PCR. Touch-down PCR thermocycler conditions were adapted to provide homogenous amplification of PCR templates of varying sizes. The purified and quantified PCR pools were diluted to a concentration of 4 nM, and 3 μl of this dilution from each of the 48 MIDs were pooled together into one final pool for next-generation sequencing (NGS).

NGS was performed using the MiSeq platform (Illumina, California, USA) and Reagent Kit v2 (500 cycles) cartridge (Illumina) according to the manufacturer’s instructions. Sequencing was carried out with 250 bp paired-end sequencing. After the run, the sequencing reads were processed by Illumina Genome Analyzer. FASTQ files were analyzed further using the Amplikyzer2 software [47], which can calculate methylation rates at both single nucleotide and regional levels. To determine allele-specific methylation rates, the analyzed *BEGAIN* sequence was aligned to the reference genomic sequence and allele splitting was based on SNP rs7141087. Only reads with an overall bisulfite conversion rate of >95% were considered and further downstream processing of Amplikyzer output files was performed.

### Statistical and bioinformatic analysis

Statistical analysis was performed using IBM SPSS version 28. The parental age in FCBs was correlated with the DNA methylation levels at the individual CpG and the regional level. Depending on the data distribution, either Pearson’s or Spearman’s correlations were used. Parametric *t*-tests or non-parametric Mann-Whitney *U*-tests were performed for group comparisons. A *p*-value of < 0.05 was considered as statistically significant throughout the analyses. Regression analysis of BPS and DBS data has been performed using the “lm” routine of the statistical software package R (version3.6.3) and visualized with the “crPlot” routine of the car package. Reanalysis of a genome-wide DNA methylation data set (GSE53924) on post-mortem human brain samples from individuals with and without ASD [20] was performed using the GEO2R tool. Here we focused exclusively on male samples, including 8 autistic individuals and 10 controls to investigate the *BEGAIN* methylation status of a CpG site in the target gene region of our BPS assay. Bulk RNA-Seq gene expression for the *BEGAIN* gene (ENSG00000183092.16) was visualized using the GTEx portal (https://www.gtexportal.org/home/).

## Supplementary Material

Supplementary Figures

Supplementary Table 1
