# Supplementary material for: Effects of paternal and chronological age on BEGAIN methylation and its possible role in autism
Source: Aging (Albany NY). 2023 Nov 28;15(22):12763–79. doi: 10.18632/aging.205275 (PMC10713433; doi:10.18632/aging.205275)
Supplement: Supplementary Figures [file aging-15-205275-s001.pdf]

SUPPLEMENTARY FIGURES

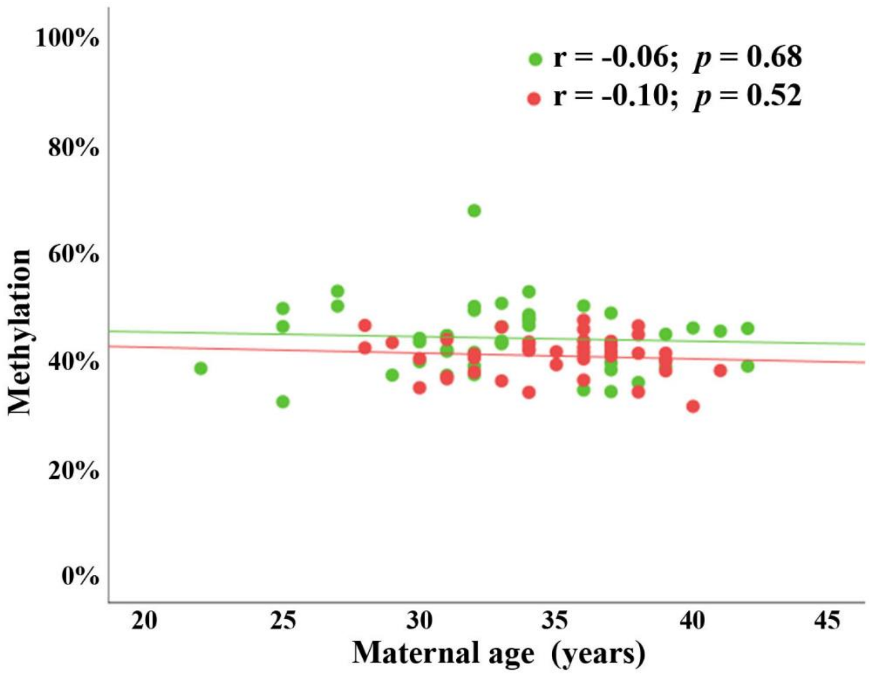

Supplementary Figure 1. No detectable maternal age effect on cord blood methylation. Methylation of the human *BEGAIN* promoter (y-axis in %) is not affected by maternal age (x-axis in years). Red dots represent 43 male and green dots 46 female FCB samples.

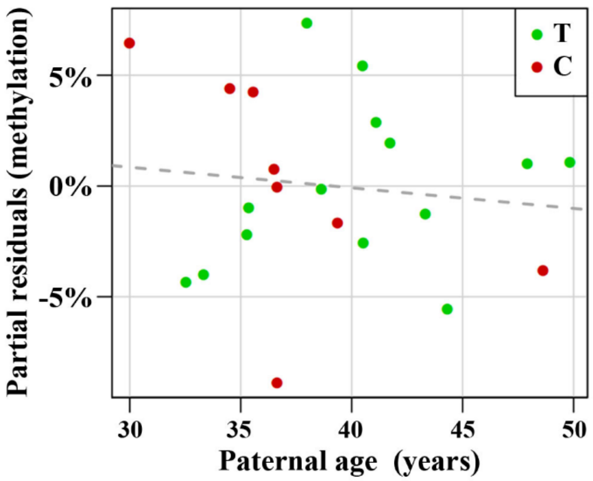

Supplementary Figure 2. Paternal allele methylation of the human *BEGAIN* promoter in male cord bloods. The component and residual plot shows a negative association (coefficient of regression model = -0.09; $p = 0.62$) between paternal age (x-axis in years) and mean methylation of 14 CpGs (y-axis in %) of the paternal allele in FCB samples. Green dots represent FCBs with a paternal T and red dots samples with a paternal C allele. The partial residuals are adjusted for the base effects of SNP rs7141087.

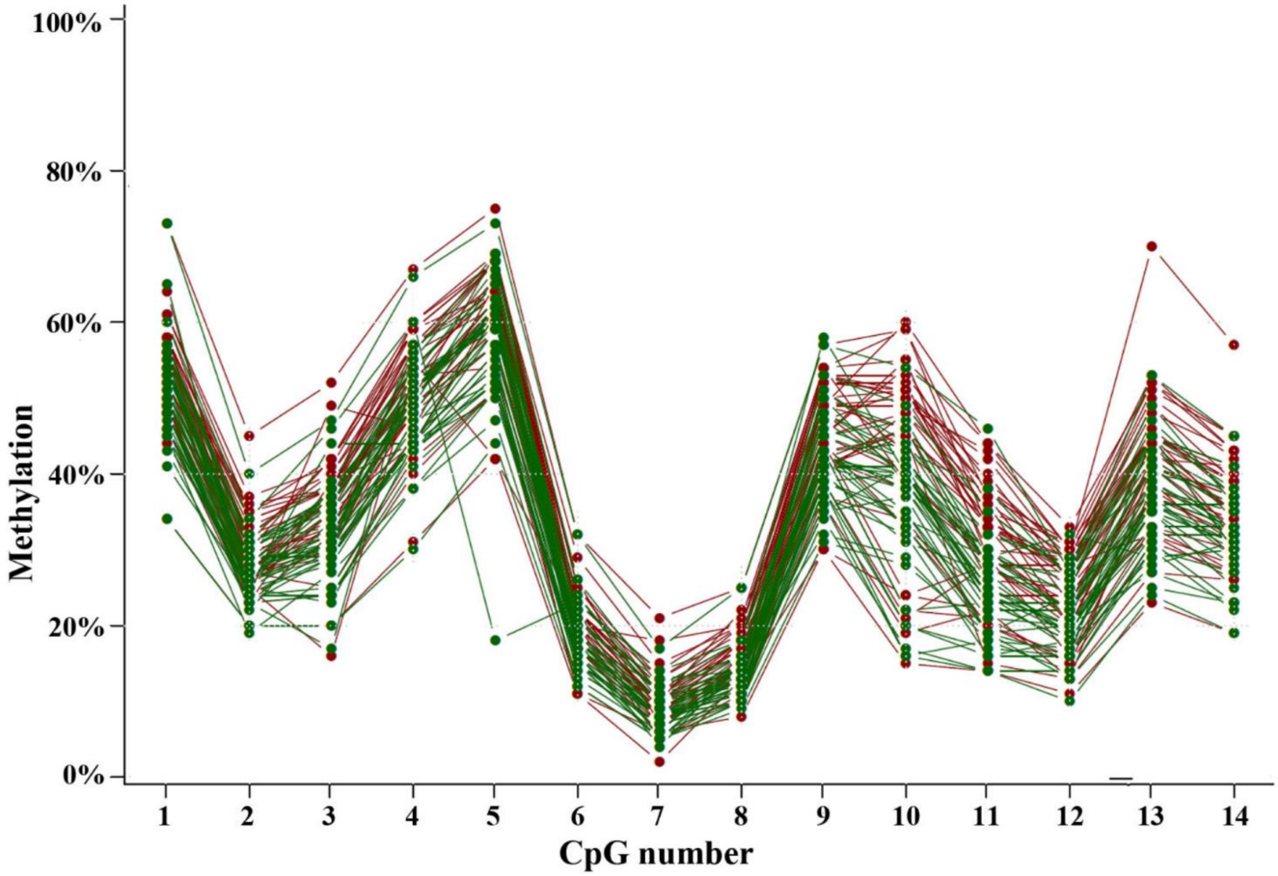

Supplementary Figure 3. No evidence for parent-specific *BEGAIN* methylation in FCBs. The x-axis indicates 14 contiguous CpG sites in the *BEGAIN* promoter region studied by DBS, the y-axis the methylation level at a given site. Thin colored lines represent methylation profiles of individual alleles. Green lines represent the maternal and red lines the paternal alleles of 38 informative samples.

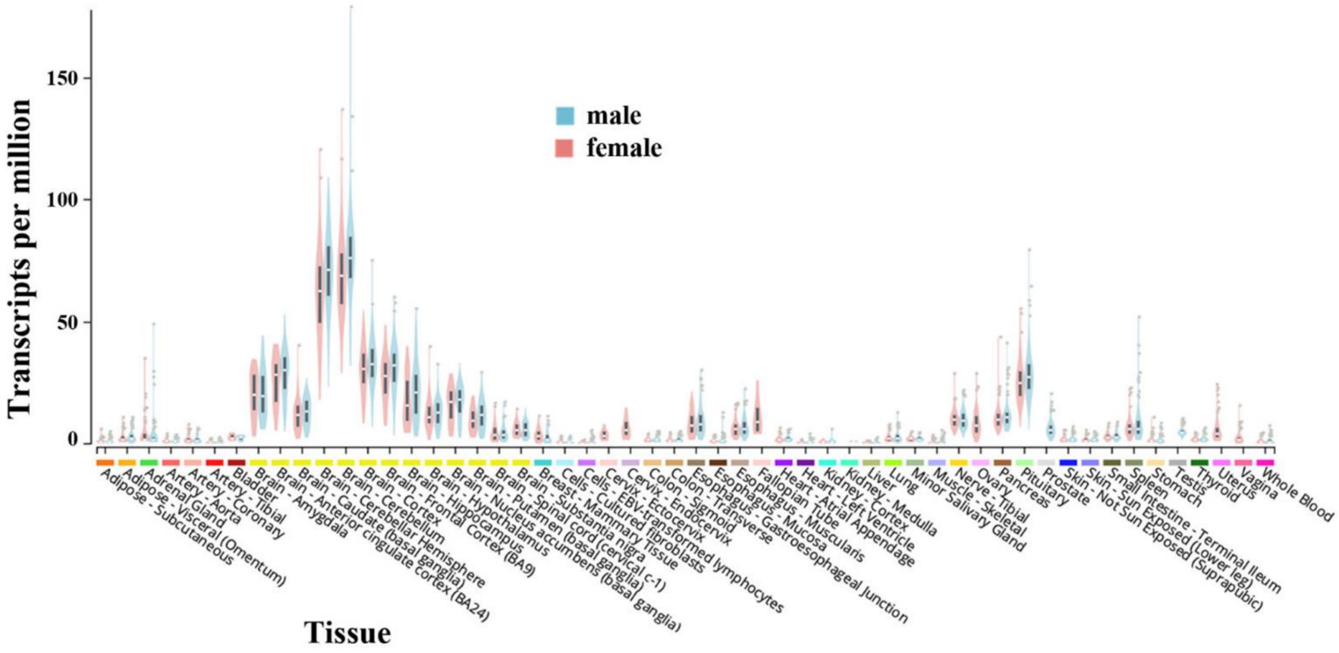

Supplementary Figure 4. *BEGAIN* gene expression across different human tissues using the GTEx data set. The y-axis displays the expression values in transcripts per million (TPM). The box plots are displayed as 25th and 75th percentile with the horizontal white line indicating the median. Outliers above or below 1.5 times the interquartile range are displayed as points. *BEGAIN* is widely expressed in the brain with the highest expression in cerebellum and cerebellar hemisphere. Please note that in most brain regions *BEGAIN* expression is somewhat higher in males (light blue bars), compared to females (pink bars).

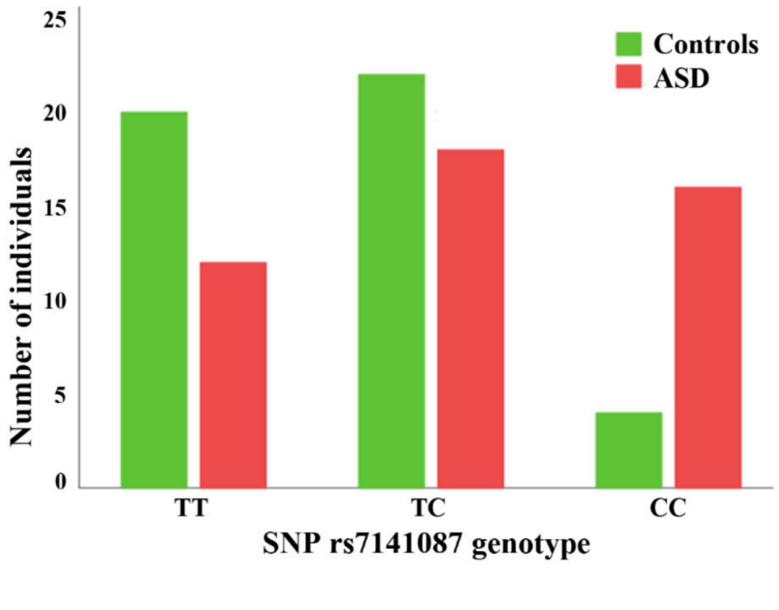

Supplementary Figure 5. Genotype distribution of SNP rs7141087. Bar diagrams showing the number of TT, TC, and CC genotypes in 40 individuals with ASD (red bars) and 40 age- and sex-matched controls (green bars). Please note that the CC genotype is overrepresented in the ASD group.
