# Supplementary material for: Effects of paternal and chronological age on BEGAIN methylation and its possible role in autism
Source: Aging (Albany NY). 2023 Nov 28;15(22):12763–79. doi: 10.18632/aging.205275 (PMC10713433; doi:10.18632/aging.205275)
Supplement: Supplementary Table 1 [file aging-15-205275-s002.pdf]

SUPPLEMENTARY TABLE

Supplementary Table 1. PCR and sequencing primers (for bisulfite converted DNA) of the human *BEGAIN* promoter region.

Technique	Primer	Sequence (5'-3')[a]	Location (GRCh 38)	Annealing Temp. (° C)	Target
Pyrosequencing	Forward	GTTTTGTTTTTTAGGGGTTAATGAGGA	Chr 14: 100,569,818 – 100,570,039	60° C	
	Reverse	*AAATCTCCAACAAACCTCTTCTCTAT			
	Sequencing 1	GGGTTAATGAGGAAAATTTTT			2 CpGs
	Sequencing 2	AGGTTATTTTTAGTAGAATGG			5 CpGs
	Sequencing 3	AGTTTTGTTATGGAAGTTT			2 CpGs
Genotyping	Forward	*TTGTTGGAGAATTTAGTTTAGAGTTAG	Chr 14: 100,569,607 – 100,569,771	60° C	SNP rs7141087
	Reverse	ACACCCAACAAACTTAACCTAC			
	Sequencing	AACCCAAAAAAATCCAAATA			
DBS	Forward	TAGTAAGTTTTTTTTTTGTTGGAGTTT	Chr 14: 100,569,573 – 100,570,032	60° C	14 CpGs
	Reverse	ACAAAAACCCTATATTCTCACCAAAACAC			

[a]Primers indicated by a star are biotinylated at the 5' end.
